# Towards Semantically Sensitive Text Clustering: A Feature Space Modeling Technology Based on Dimension Extension

**DOI:** 10.1371/journal.pone.0117390

**Published:** 2015-03-20

**Authors:** Yuanchao Liu, Ming Liu, Xin Wang

**Affiliations:** School of Computer Science and Technology, Harbin Institute of Technology, Harbin, China; University of Memphis, UNITED STATES

## Abstract

The objective of text clustering is to divide document collections into clusters based on the similarity between documents. In this paper, an extension-based feature modeling approach towards semantically sensitive text clustering is proposed along with the corresponding feature space construction and similarity computation method. By combining the similarity in traditional feature space and that in extension space, the adverse effects of the complexity and diversity of natural language can be addressed and clustering semantic sensitivity can be improved correspondingly. The generated clusters can be organized using different granularities. The experimental evaluations on well-known clustering algorithms and datasets have verified the effectiveness of our approach.

## Introduction

In an effort to cope with the tremendous growth of electronic documents on the World Wide Web, many studies have investigated how to organize such information in a way that will make it easier for the end users to find the information they request efficiently and accurately. Since most available online resources are text-based, the ability to efficiently organize massive text data so that it is easily searched and explored is in demand. One way to fulfill such requirements is text-clustering technology, and it has received special and increased attention from researchers in the past few decades [1] [2]. Clustering technology, particularly accurate clustering, plays an important role in many applications including the automatic organization of complex text information, information retrieval, and question answering.

The objective of text clustering is to divide document collections into clusters based on the similarity of features. These clusters should have high intra-cluster similarity and low inter-cluster similarity [3]. Clustering data on a hierarchical structure or into different clusters enables exploring data on different levels of granularity, providing a more intuitive view that is close to the way humans view the world [4]. Researchers have found that documents in a collection naturally lend themselves to a hierarchical structure [5]. Thus a clustering technique with different granularity is powerful for preliminary analysis of text data. In fact, many clustering algorithms allow people to set clustering granularity, such as the k value of k-means [6] and the number of neurons on output layers of SOM (Self-Organizing Maps) [7].

In order to produce high-quality text clustering results with different granularity, the semantic factor is much more important. Basically, bigger granularity means the number of clusters in clustering algorithm (e.g., the k value of k-means) is smaller, thus each cluster may be bigger as they usually contain more documents; whereas smaller granularity means the number of clusters in clustering algorithm is bigger, thus each cluster may be smaller as they usually contain less documents. When observing documents with bigger granularity, it becomes difficult for a text clustering system to grasp the common features between documents that should be assigned to one bigger cluster, as many common features may be hidden by complex language expressions [8].

The current feature modeling theories and methods for text clustering remain limited, given that subtle semantic issues cannot be grasped and coded. For text mining tasks, the majority of state-of-the-art frameworks employ VSM (Vector Space Model) [9] and their variants [10] [11], which treat a document as a bag of words and use plain language words as features. Although this type of model is able to represent text mining problems easily and directly, it suffers from the problems of polysemy and synonymy. This may be especially true for short and sparse documents (e.g., search result snippets, product descriptions, and book/movie summaries), because these short documents do not provide enough word co-occurrence patterns or shared contexts to obtain a useful similarity measure. Therefore, current feature coding technologies based solely on term frequency usually fail to achieve the desired accuracy due to data sparseness.

In this paper, we propose a novel feature space modeling method based on semantically sensitive text clustering. All documents are represented in two feature spaces: one is the traditional space; the other is the extension space. We use a lexicon chain to find the hidden sub-topics in each document and to extract the basic features and construct the traditional feature space, and then these basic features are semantically extended to construct the extension feature space. By combining both the similarity in traditional space and that in extension space, our clustering system can improve clustering semantic sensitivity and address the adverse effects caused by the complexity and diversity of natural language. The generated clusters are easily organized into a cluster hierarchy with different granularities. Through this method, the documents that belong to the same general topic but share few common words will have higher similarity with each other, thus the clustering accuracy will be improved even when the granularity is bigger, and our system can successfully unveil inherent structures in the underlying text documents. We believe that our approach is suitable for applications where it is necessary to observe documents, especially those that share few surface features among the intra-cluster documents, with different levels of granularity.

The rest of this paper is organized as follows. In Section 2, we describe prior related studies. Sections 3 and 4 discuss the strategy of semantic extension at the word level and at the document level separately. In Section 5, the comprehensive similarity computation method based on semantic extension for text clustering is presented. Then, in Section 6, the experiments and evaluation results are explained and discussed. Finally, Section 7 discusses the conclusions and future studies.

## Related Work

### 2.1 Document Representation Model

The bag-of-words (BOW) representation is commonly used for text classification/clustering tasks. Features are usually chosen from the terms in the documents. After the feature space is constructed, each document is mapped to this space and represented as one high-dimension vector in the feature space. Despite this high-dimension representation form, text clustering still faces the problem of low semantic sensitivity due to the complexity of natural language processing.

Feature selection techniques are used to identify the most suitable terms. Some feature selection technologies, such as LSI (Latent Semantic Indexing) [12], provide a mathematical tool to project high-dimensional data into lower dimensional subspace through algebraic transformations. Thus the documents can be represented on a semantic level rather than by lexical occurrence. LDA is also closely related to the probabilistic latent semantic analysis as proposed by Hofmann [13], and is a probabilistic formulation of LSA. LDA has been successfully used for processing large collections [14] [15]. It is still difficult for LDA and LSI to reflect the rich world knowledge using only algebraic transformations on the document collection to be processed. There are also other feature selection algorithms, e.g., IG (Information Gain) and CHI Square analysis that are widely used in text processing, but finding a globally optimal solution is often an NP-hard problem [16].

### 2.2 Similarity Computation

The similarity computation between documents or that between documents and clusters appears frequently in text clustering. Some of the more common distance measures in text clustering include the Euclidian distance [17] and the tf-idf cosine similarity [18]. Among them, cosine similarity is a popular function for distance/similarity measure, especially for text applications. There are also other distances for specific uses, such as the Kullback—Leibler (KL) distance [19] and the Jensen-Shannon (JS) divergence [20].

Besides, Metzeler et al. [21] evaluated a wide range of similarity measures for short texts. Gabrilovich and Markovitch [22] computed semantic relatedness using Wikipedia concepts that are widely available using most commercial search engines.

### 2.3 Concept-based Clustering

Some efforts have been targeted toward concept text clustering analysis [23] [24]. Shehata et al. [25] proposed a concept-based similarity measure that is capable of calculating similarity of pairwise documents by combining the factors affecting the weights of concepts on sentence, document, and corpus levels. Their studies demonstrate that the newly developed concept-based mining model enhances the clustering quality of sets of documents substantially. Inspired by the observation that text documents contain semantically coherent sets of ideas/topics, Ji fully studied document clustering when prior knowledge is utilized [26]. Besides, there is also related work that includes concept chain-based text clustering proposed by Song [27], a semantic smoothing clustering model proposed by Liu [28], and a novel text clustering algorithm based on an inner product space model of semantics proposed by Peng [29].

Although there are some reports about the semantic role in text clustering, the study that has been reported in the literature about semantic extension in vari-granularity (vari-granularity means variable or varying granularity, e.g., different k values in k-means) text clustering is limited. With the variance of granularity, the feature coding method usually affects text-clustering quality. The granularity in many clustering algorithms can be adjusted; therefore, people can observe these documents from different angles. For example, when the k value of k-means is smaller, the granularity is also bigger. Different from clustering of other data types, when bigger granularity is set for text clustering, it becomes difficult for many traditional coding schemes to place documents about the same topic together. This indicates that text clustering is significantly different from clustering of other data types.

## The Semantic Extension at Word Level

**Fig 1:**
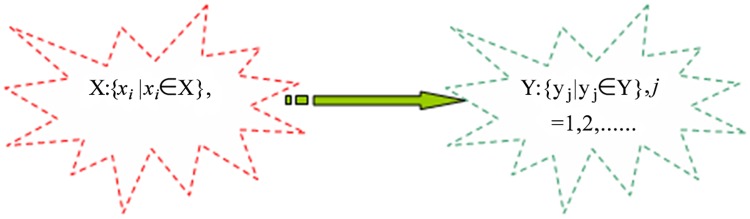
The extension at the word level.

As shown in Fig. 1, suppose X and Y are two word sets, X is the keyword set extracted from one document, and for each word element *x*_*i*_ in X, there is one corresponding word element *y*_*j*_in Y, where *y*_*j*_ includes the semantic features of word *x*, i.e., *y*_*j*_may be similar (a synonym) or is related to *x*_*i*_ *x*_*i*_, or may be an upper concept word of *x*_*i*_. Here, the mapping of *x*_*i*_->*y*_*j*_ means the semantic extension at the word level to capture the common features of each word.

Generally, a synonym means a different expression for the same meaning; for example, the terms “*kid*” and “*child*” are synonyms. Abbreviations can also be deemed as synonyms; for example, “*DNA*” is the abbreviation for “*deoxyribonucleic acid*”. Although people can easily understand that they have in fact one meaning, it will be difficult for a clustering system to recognize synonymous relationships if this knowledge is not provided. In most situations, one word can be replaced by its synonym in its context. Correlative words are those words with a close relationship to each other, and usually belong to one small field. For example, “*computer*,” “*disk*,” and “*keyboard*” are correlative words. Another phenomenon to be processed may be the multi-level concept mode. For example, terms like “*Mars*,” “*Venus*,” “*Mercury*,” “*Uranus*,” and “*Polaris*” can all be mapped as the word “*planet*,” because the latter is their upper-level word and they are all types of planets. In terms of an actual text clustering system, if one document is about Mars, another is about Mercury, and they share few common words, both documents will be separated into different clusters by most text clustering systems. In order for a clustering system to partially “understand” semantic information, such knowledge must be provided to a computer in an effective way.

### 3.1 Extensions using world knowledge

As the knowledge sources, *CILIN* [30] and *HOWNET* [31] can provide rich world knowledge support for similarity computation. *CILIN* is a synonym lexicon. The words in *CILIN* involve 3 levels: 12 big classes, 94 middle classes, and 1,428 small classes. Below the small classes, there are also various word groups. For each word group there is one title that usually contains some of the most frequently used words in that group. There are 3,925 titles in total in *CILIN*. When using *CILIN* as a knowledge source of text clustering, the semantic structure of *CILIN* and the practical needs of text clustering should both be taken into consideration. Here is an example demonstrating the 3 different levels of class for word “*infant*” from *CILIN*.

**Example**: Small-class title of word “infant” is “Ab04 kid, baby, and child”. Its middle-class title is “Ab man, woman, older, younger” and its big-class title is “A human.”

It can be seen that the small-class title of *CILIN* can closely represent the common features of words in it and can be helpful for text clustering, whereas the middle-class title and big-class title seem too wide. Therefore, in this paper, the mapping of words using small class *CILIN* titles will be utilized for extension. The process is shown in the following steps:

*Algorithm 1*. *Semantic extension using CILIN*

*BEGIN*

 [*1*]. *For each keyword xi from the keyword set X of the documents to be processed*;

 [*2*]. *Search the small-class title in CILIN*;

 [*3*]. *If found, denote its asyj*;

 [*4*]. *Putyj intoY',which is the CILIN extension keyword set of document D*;

 [*5*]. *Else*, *continue*;

 [*6*]. *End for*;

*END*

Compared with *CILIN*, *HOWNET* can provide additional support for text clustering. This is because *HOWNET* is a networked knowledge system, in which the relationships among concepts and the relationships among properties are labeled. This is consistent with text clustering, as the objective of a text clustering system is to bring closely related documents together. In most situations, the relevance of documents lies in the relevance of words. If the content words in different documents are very close, then these documents will also be as much closer.

Similar to *CILIN*, there are 3 relevance levels in *HOWNET*: Levels 1, 2, and 3. Level 1 is a closer relevance level than the other two levels. When the level is determined, all the relevance words can be found. In terms of text clustering, we select level 1 in this paper. In *HOWNET*, all relevance words are symmetrical, thus we can select the first word in the relevance set as the representative word for all words in the relevance set, and the mapping is clearly that of many-to-one. For one word, there may be some different “synsets” in *HOWNET*. A synset contains a set of synonymous words for a particular sense of a word. Thus it is necessary to disambiguate the senses before extension. One simple way is by selecting the meaning *s*_*M*_(*x*_*i*_)that has the biggest intersection between the keywords of the original document and all the relevant terms of sense *s*_*k*_(*x*_*i*_). This creates a “meaning” record that will be the index to find the representative words for the extension space. Therefore, the extension process using *HOWNET* is shown in the following steps:

*Algorithm 2. Semantic extension using HOWNET*

*BEGIN*

 [*1*]. *For each keyword xi from the keyword set X of the documents to be processed*;

 [*2*]. *Search for xi in HOWNET*;

 [*3*]. *If found, select the meaning sM(xi)that has the biggest intersection between the keywords of the original document and all the relevant terms of sense sk(xi)*;

 [*4*]. *Else*, *continue*;

 [*5*]. *Select the first relevant term from sM(xi)of xi as yj*;

 [*6*]. *Put yj into Y*", *which is the HOWNET extension keyword set of document D*;

 [*7*]. *End for*

*END*

With algorithms 1 and 2, *Y'*and *Y"*of one document *D* can be obtained as the extension keyword set *Y*(*Y* = *Y'*∪ *Y"*) that can be further used to construct the extension feature space. Through the above mapping, the documents for the same topic can be easily brought together because most of their extension words may be the same ones, even if they share few common words. Besides, some keywords of same documents may be mapped as the same extension word, allowing frequency information to be counted and the final semantic numerical value for these features will be strengthened.

### 3.2 Extension using Latent Dirichlet Allocation

Latent Dirichlet Allocation (LDA) is a widely used generative Probabilistic topic model in recent years. It assumes that each document is a mixture of a small number of difficult topics and that each word's creation is attributable to one of the document's topics. A lexical word may occur in several topics with a different probability.

**Fig 2:**
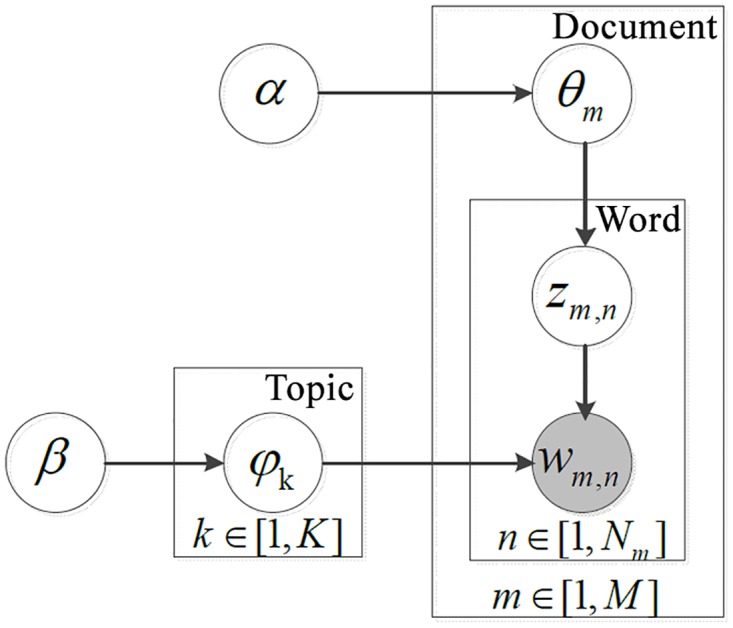
The LDA model.

LDA can help find the latent topic distribution in the text collection to some extent, thus it may provide knowledge about the text collection and some world knowledge. The generation principle of LDA can be depicted in Fig. 2.

In Fig. 2, M is the number of all documents, K is the topic number, V is the vocabulary size, *N*_*m*_ is the number of words for document *m*, *w*_*m*,*n*_, *z*_*m*,*n*_ means the words observed and the corresponding hidden topic variable for the position *n* in document *m*. *θ*_*m*_denotes the k-dimension Multinomial Distribution for document m. *α* is the parameter of the Dirichlet prior on the per-document topic distributions, *β*is the parameter of the Dirichlet prior on the per-topic word distribution. *ϕ*_*k*_ is the word distribution for topic *k*.

In this paper, we use The Gibbs sampling [32] to train the LDA model. By estimating the LDA parameters using Gibbs sampling, we can get the topic distribution of documents. The sampling probability is

$$p(z_{m',n'}=k|Z_{\neg z_{m',n'}}^{new},w_{m',n'}=v,W_{\neg w_{m',n'}}^{new};\Psi_{k,v})=\frac{\Psi_{k,v}+\Psi_{k,v}^{new}+\beta_{w_{m',n'}}-1}{[\sum_{v’=1}^{V}\left(\Psi_{k,v’}+\Psi_{k,v}^{new}+\beta_{v’}\right)]-1}\cdot \frac{\Omega_{m',k}^{new}+\alpha_{k}-1}{[\sum_{k=1}^{K}\left(\Omega_{m',k}^{new}+\alpha_{k}\right)]-1}$$ (1)

Here Ψ_*k*,*v*_ is the sampling result on the original text collections. $\Omega_{m',k}^{new}$ means the count of topic *k* shown in document *m*'. $\Psi_{k,v}^{new}$ means the count of word v in document collection under topic *k*. After iteration, the topic distribution of new document can be denoted as

$$\theta_{m',k}=\frac{\Omega_{m',k}+\alpha_{k}}{\left[\sum_{k=1}^{K}\left(\Omega_{m',k}+\alpha_{k}\right)\right]}$$ (2)

In this way, the topic distribution of text collection can be estimated, which we can use for similarity computation, as shown in section 5.

## The Extension Strategy at the Document Level

In Section 2, the method of how to obtain the semantic extension Y of one document D from the keyword set X has been provided. In this section we will introduce the document keyword extraction method, and discuss the different possibilities of the combination of two similarity computations by using both X and Y.

To achieve better clustering results, the data representing model must accurately capture the salient features of data. Selecting features from text documents is one of the key issues in text clustering/classification tasks and has attracted a fair amount of research with various methods proposed [33][34]. The widely used VSM (Vector Space Model) is simple and convenient for computation, but the relationships between words are neglected and its practical application performance is limited. According to VSM, each document is represented as a feature vector of terms with different weights assigned to the terms according to their frequency of appearance in the document. It does not represent any relationships between the words. To address this problem, we use lexicon chain technology [35] to extract the keywords in different semantic chains from the document. This is because lexicon chain provides the representation of a document’s structure, and produces the important outline of the semantic structure, thus it is also helpful for text clustering that attempts to capture the main topic of documents or document collections.

**Fig 3:**
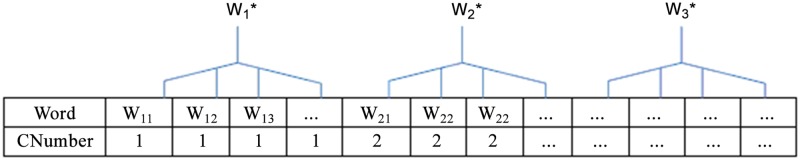
Selection of keywords by using lexicon chain technology (CNumber is the index of different lexicon chains).

Through text clustering technology, the outline of a collection of documents can be captured. By analyzing the lexical chain of each document, the outline of each document is captured and represented by extracting the important keywords in different semantic chains. In Fig. 3, we demonstrate the basic principle of keyword selection by using a lexicon chain. As shown in Fig. 3, w1*, w2*, w3*…, and etc. can represent the keywords extracted from different chains in one document. The Lexicon Chain Construction steps are shown as algorithm 3.

*Algorithm 3*. *Lexicon Chain Construction*

*BEGIN*

 [*1*]. *Initialize chaincount* = *0*;

 [*2*]. *Segment document D into a term set S*;

 [*3*]. *Stop word removal of S*, *form S’*;

 [*4*]. *For each term W in S’*;

 [*5*]. *For each chain C*;

 [*6*]. *If W is related to C*, *add W to C*;

 [*7*]. *Otherwise*, *screate a new chain C’*, *and add W to C’*;

 [*8*]. *Chaincount* = *chaincount* + *1*;

 [*9*]. *End for*;

 [*10*]. *End for*;

*END*

In order to judge If W is related to C in Step 6 of algorithm 3, we use *HOWNET* as the knowledge base. In practice, we only keep the lexicon chains that have at least two words in it. In this way, salient features will be kept and non-salient ones will be filtered. Basically all these keywords in salient chains should be used as keywords and extended in this paper, as shown in Section 3. It is also critical to note that in this way only a few words for each document need to be extended and because there are many documents to be processed and some keywords from these documents may be the same, the size of the extended word set is much smaller.

**Fig 4:**
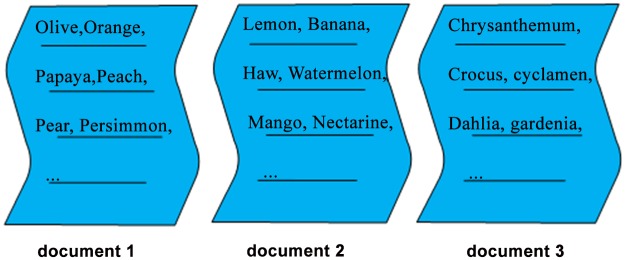
An example for demonstrating the effect of semantic extension on document level.

Fig. 4 is a brief example which explains the function of semantic extension. In Fig. 4, there are 3 documents. Both document 1 and document 2 are about the topic of some fruits and they should be put into one cluster, whereas document 3 is about the topic of flowers and it should form a separate cluster. But it is usually difficult for text clustering system to achieve this goal because document 1 and 2 have no intersection, i.e., they share no apparent surface features. In particular, if k value is set to 2, document 1 or 2 may be wrongly put into the cluster where document 3 resides in some occasions. This problem can be solved by semantic extension as there are underlying semantic relations between document 1 and 2.

## Similarity Computation

Through the extension methods described in Sections 3 and 4, the keyword set and the extension keyword set for each document can be obtained. In this way, besides the traditional word vector, an extension vector is added, thus an extension feature space for text clustering will also be needed. All documents are represented in these two spaces and the overall similarity is determined from their combination.

Through the introduction of semantic extension technology, the relationships between documents are measured in two feature spaces: one is the widely used VSM space; the other is the extension space. The extension space will help to capture the common features hidden from the surface features for text clustering, a problem that is difficult to overcome by traditional methods.

In fact, these two spaces can compensate for each other. For example, according to our scheme, the extension vector of two totally different documents may be the same, but the two documents may in fact contain some slight differences in hidden common features. If the traditional document representation model were not used, the documents would be classified as dissimilar, so the traditional method is also necessary and should be taken into consideration.

Based on the above considerations, the similarity calculation between documents is based on a combination of traditional similarity and extension-based similarity. Thus the comprehensive similarity *Sim*(*d*_*1*_,*d*_*2*_) between *d*_*1*_and *d*_*2*_ can be expressed as follows:

$$Sim(d_{1},d_{2})=(1-\alpha )*Sim_{1}(d_{1},d_{2})+\alpha *Sim_{2}(d_{1},d_{2})$$ (3)

In formula (3), *Sim*(*d*_*1*_,*d*_*2*_)and *Sim*(*d*_*1*_,*d*_*2*_)can be calculated by cosine similarity as follows:

$$Sim_{1}(d_{1},d_{2})=\frac{\sum_{k=1}^{m}W_{1k}\times W_{2k}}{\sqrt{\left(\sum_{k=1}^{m}W_{1k}^{2})(\sum_{k=1}^{m}W_{2k}^{2}\right)}}$$ (4)

$$Sim_{2}(d_{1},d_{2})=\frac{\sum_{k=1}^{n}W_{1k}\times W_{2k}}{\sqrt{\left(\sum_{k=1}^{n}W_{1k}^{2})(\sum_{k=1}^{n}W_{2k}^{2}\right)}}$$ (5)

In formula (4) and (5), the former similarity *Sim*(*d*_*1*_,*d*_*2*_) is the similarity in traditional VSM space; the latter similarity *Sim*(*d*_*1*_,*d*_*2*_) is the similarity in extension space. *m* and *n* are the dimension numbers of these two different feature spaces, respectively. Here, *α* is used as the blend factor. *α* = 1means only the latter similarity is used, whereas *α* = 0 means only the former similarity is used. Thus it can be seen that our model is compatible with the traditional model. Note that *α* can be used as an important factor for vari-granularity text clustering; as we will see later, when granularity is bigger, more semantic extension knowledge is added, so the value of *α* must be set higher to utilize more extension knowledge.

When LDA model is used for extension instead, *Sim*_*2*_ can be represented as the vector in topic space. *d*_*LDA*_ = {*t*_*1*_,*t*_*2*_,…,*t*_*k*_}. Here k means the topic dimension. Then the similarity between two documents is *Sim*_*2*_ *= Sim*_*LDA*_(*d*_*i*_,*d*_*j*_).

## Experimental Results and Discussion

### 6.1 Experiment Setting and Evaluation Measures

We conducted a set of experiments using our proposed semantic extension model and similarity measure. In our approach, we use hard clustering, where only one cluster was assigned to each input document. In addition, the total number of clusters required is pre-specified, as we focused on the semantic modeling ability of our method. There are two datasets used in this paper. Dataset 1 is downloaded from web site, http://news.sina.com.cn. There are 10 different main/big topics: “pets,” “domestic life,” “cuisines,” “automobile,” “child rearing,” “politics,” “military affairs,” “sports,” “tourism,” and “aerospace.” Each topic contains 5 sub-topics, and each sub-topic contains 20 documents; therefore, there are in total 1000 documents. Although generally speaking the documents in the same sub-topic share more common words, the common words are fewer and sparser for different sub-topics in one big topic. We designed the dataset in this manner so that the effect of semantic feature space modeling on vary-granularity text clustering can be evaluated objectively. Dataset 2 is the well-known dataset 20 Newsgroups [36] text corpus (we use *HOWNET*, which is Chinese/English lexicon, to make the semantic extension for dataset 2). The reason for choosing 20 Newsgroups is that they are also in fact a hierarchy text collection. For example, both “rec.sport.baseball” and “rec.sport.hockey” are about sports; whereas “talk.politics.guns,” “talk.politics.mideast,” and “talk.politics.misc” are about politics, thus they represent one big topic. In Table 1, we summarize the basic information about datasets 1 and 2.

**Table 1:** The two datasets.

No.	#of keywords	k1	k2	# of documents	Source
1	4296	10	50	1000	http://news.sina.com.cn
2	63204	8	20	19997	20 Newsgroups

In many applications, the number of clusters to be generated depends on the granularity that people view these documents. In this paper, we suppose people look from two different viewpoints: high level and low level. Thus, the *k* value, i.e., the number of clusters, is set to be 10 and 50 (k1 = 10, K2 = 50) in dataset 1, whereas 8 and 20(k1 = 8, K2 = 20) separately in dataset 2.

To evaluate the performance, we compare the clusters generated by semantic clustering modeling methods with the category distribution. We first evaluate our system quality by accuracy. The accuracy of each cluster is defined as the number of documents of the major class in a cluster divided by the total number of documents in that cluster. The major class of one cluster has the biggest number of same-class documents within that cluster. The overall accuracy of one clustering is then calculated as the sum of the average value for each cluster.

$$Accuracy(Cluster_{i})=\frac{\#ofdocuments(MajorClass(i)\cap Cluster_{i})}{\#ofDocuments(Cluster_{i})}$$ (6)

$$OverallAccuracy=(\sum_{i}Accuracy(Cluster_{i})/(\#\;of\;Clusters)$$ (7)

Another clustering evaluation measures is to compute the BCubed F-Measure [37] [38]. Suppose *L*(*i*) be the category of document *i* and *C*(*i*) be the cluster of document *i*, then relation correctness between document *i*and document *j* can be calculated as:

$$Correctness(i,j)=\left\{\begin{array}{l} 1,if\;L(i)=L(j)\;and\;C(i)=C(j)\\ 0,\;otherwise \end{array}\right\}$$ (8)

Then the BCubed precision *BP*_*i*_ of document *i* can be defined as the percentage of the number of correct documents in cluster containing document *i* and the number of documents in cluster containing *i*. The BCubed recall *BR*_*i*_ of document *i* can be defined as the percentage of the number of correct documents in output cluster containing *i* and the number of documents in the category containing *i*.

The overall BCubed precision *BP* and BCubed recall *BR* can be defined as

$$BP=(\underset{i}{\sum }BP_{i})/(\#\;of\;all\;documents)$$ (9)

$$BR=(\underset{i}{\sum }BR_{i})/(\#\;of\;all\;documents)$$ (10)

Then BCubed F-Measure is the combination of *BP* and *BR*:

BF=(2*BP*BR)/(BP+BR) (11)

In the following, we first demonstrate the effectiveness of our model by comparing clustering results for different granularity and different similarity factor for different clustering methods, and then we make a comparison of time consumed with or without semantic extension on these datasets.

### 6.2 Clustering performance for different granularity and different blend factor

**Fig 5:**
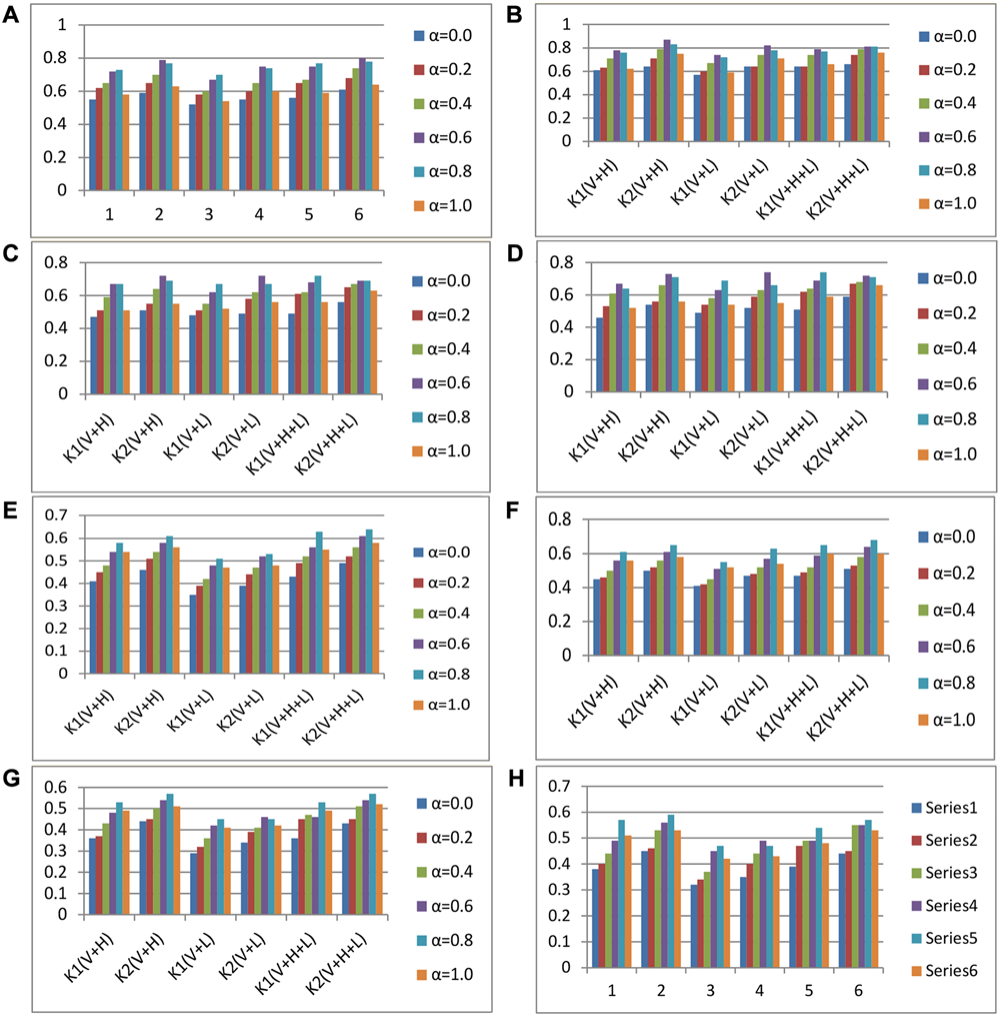
Clustering performance for different granularity and different blend factor. (A). Evaluation method: accuracy; Clustering method: k-means; Dataset: dataset 1; (B). Evaluation method: accuracy; Clustering method: SOM; Dataset: dataset 1; (C). Evaluation method: BF; Clustering method: k-means; Dataset: dataset 1; (D): Evaluation method: BF; Clustering method: SOM; Dataset: dataset 1; (E). Evaluation method: accuracy; Clustering method: k-means; Dataset: dataset 2; (F). Evaluation method: accuracy; Clustering method: SOM; Dataset: dataset 2; (G). Evaluation method: BF; Clustering method: k-means; Dataset: dataset 2; (H). Evaluation method: BF; Clustering method: SOM; Dataset: dataset 2.

To better understand the actual effect of semantic extension on clustering quality and its application for vari-granularity clustering, we generated a clustering quality profile using different similarity blend factors, and different granularities for two clustering algorithms: k-means, and SOM separately in Fig. 5(A)–(D) and Fig. 5(E)–(H). These two clustering algorithms are widely studied in related studies. Therefore, we choose them to examine the effectiveness of our approach. We also make comparisons for different semantic modeling methods: VSM+HOWNET, VSM+LDA, VSM_HOWNET+LDA on different granularities, the results are demonstrated in these figures (abbreviated as V+H, V+L, and V+H+L correspondingly). We use GibbsLDA++ (http://gibbsLDA.sourceforge.net) to train the topic distribution of LDA (In the experiment, parameter *α* = 1, and *β* = 0.1; iteration time is 1000; topic is 50). It is also noted that when both HOWNET and LDA is used, *Sim*(*d*_*1*_,*d*_*2*_)in formula (1) will be replaced as the average value of extension similarity of semantic extension space and LDA space.

In Fig. 5, the blend factor is the alpha parameter presented in formula (3). It is obvious that the extension similarity enhances the quality of text clustering up to a certain point and then its effect starts to reduce the quality. As we mentioned in Section 4, although extension similarity is helpful, it alone cannot capture all the similarity information between documents; thus, the traditional similarity is still required, but to a smaller degree.

Some conclusions can be drawn from above experiments:

1. When the clustering granularity is bigger, i.e., the number of clusters (or *k* value of k-means) is smaller (e.g., *k* = 10 in Fig. 5(A)–(D) and *k* = 8 in Fig. 5(E)–(H), people can observe documents from a higher level, where there are few common features between most documents inside each category. Thus the extension can play important role for clustering. As it is shown, if a bigger semantic extension similarity is used (alpha is bigger) to some extent, the clustering performance in these figures can be improved; on the contrary, when less extension knowledge is used (alpha is smaller), the clustering performances are relatively adverse.
2. For both SOM and k-means, when alpha is smaller and the *k* value is bigger, although semantic knowledge is not fully utilized, as the granularity is relatively small and the documents in the same cluster share more common words, the clustering quality evaluated on both accuracy and BF is not adversely affected; when *k* is smaller e.g., *k* = 10 in Fig. 5(A)–(D) and *k* = 8 in Fig. 5(E)–(H), semantic modeling approach plays an important role, especially when alpha is bigger, where the clustering quality increases apparently. Whereas if alpha is big, especially when alpha approaches 1.0, semantic extension plays a completely dominant role in similarity computation, and the difference between clusters with low granularity is blurred; thus, the clustering quality is lower. This further proves that the combination with traditional similarity is necessary.
3. The improvement achieved is apparent in the clustering accuracy and BF over the traditional methods as shown from these figures. The improvements shown were achieved with similarity blend factors between around 60% and 80%. It is obvious that extension-based similarity plays an important role in accurately examining the relationships between documents. It is known that our quantization technique is relatively more sensitive to complex language phenomena.
4. The clustering results of V+L based extension model are worse by comparison. However, its performance is still better than that based only on VSM, especially using bigger granularity. When using both world knowledge (HOWNET) and text collection topic distribution knowledge (LDA), best clustering quality can be achieved. This may be because that the LDA model is usually trained from the test collection, thus only inner topic distribution is considered and obtained, even though some semantic knowledge has been observed and extracted, which is helpful for text clustering to some extent.

In order to demonstrate the extension of HOWNET and LDA, two examples have also been given in Table 2 and Table 3. Table 2 is the examples of some retrieved concept relevance records by HOWNET, whereas Table 3 is the first 6 topics from 20 newsgroups dataset discovered by gibbsLDA.

**Table 2:** The examples of some retrieved concept relevance records (in part. As some phrases records have been omitted here).

Words	Some retrieved concept relevance records
“hockey”	Rank, dribble, sports, blazer, jacket, playsuit, league, sports, team, team, exercise, prolusion, roller-skating, skating, warm-up, exercising, skate, infield, outfield, baseball
“guns”	lethality, overkill, backlash, rebound, recoil, arsenal, clip, bare-handed, unarmed, bare-handed, defenceless, unarmed, recoil, nuclearcapable, antisatellite, bulletproof, nitroglycerin, nitroglycerine, trinitroglycerin
“religion”	bodhi, cathedral, church, convent, saintdom, sainthood, confess, confession, baptise, baptism, reincarnation, transmigration, samsara, cassock, dalmatic, frock, kasaya, Buddhism, Lamaism
Sirius	constellation, skylab, observatory, planetarium, interplanetary, Altair, Andromeda, Aries, Cancer, Canopus, Cassiopeia, Draco, Galaxy, Gemini, Jupiter, Libra, Lyra, Mars, Mercury, Monoceros, Neptune
motorcycle	traffic, drive, fecundity, fertility, elevation, intercontinental, motel, magnitude, automobile, boxcar, bulldozer, cable car, caboose, car, caravan, carriage, chariot, dozer, engine, jeep, limousine
medicine	covalence, covalency, valence, valency, immunity, kelp, tremella, dosage, dose, crust, anaesthesia, anaesthetize, anesthesia, anesthetize, aftereffect, banxia, broomrape, calamus, cardmom, cardoon, costusroot

**Table 3:** The examples of first 6 topics discovered by gibbsLDA.

Topics	Some words and their scores
Topic 0th:	score 0.040230, league 0.028905, lead 0.024801, quarter 0.014953, valley 0.013476, rebound 0.012819, orange 0.012491, hill 0.010357, la 0.010357, game 0.009701, grove 0.009372, christian 0.009044, goal 0.009044, garden 0.008059, host 0.007895, visit 0.007239, kennedi 0.007239, victory 0.006910, led 0.006582, ad 0.006582
Topic 1th:	women 0.035207, research 0.021928, birth 0.017980, control 0.012956, develop 0.007572, method 0.006137, grant 0.006137, universe 0.005778, delivery 0.005778, technology0.005419, reason 0.005419, contracept0.005419, panel 0.005060, materia 0.005060, differ 0.004701, promote 0.004701, superconduct 0.004701, institut 0.004701, program 0.004701, basic 0.004343
Topic 2th:	play 0.018470, helen 0.010573, fugard 0.010573, martin 0.009696, south 0.009257, artist 0.008818, miss 0.008379, road 0.005747, benson 0.005747, coast 0.005747, central 0.005308, taper 0.005308, playwright 0.004870, April 0.004431, jan 0.004431, mecca 0.004431, stage 0.004431, house 0.004431, prize 0.003992, scr 0.003554
Topic 3th:	music 0.025085, bylin 0.020851, program 0.013795, perform 0.013442, review 0.012736, dance 0.012384, symphony 0.010972, opera 0.009208, sound 0.008855, type 0.008150, local 0.007444, concert 0.006739, sud 0.006739, classic 0.006386, festiv 0.005327, passion 0.005327, headlin 0.005327, orchestra 0.004975, predict 0.004622, director 0.004622
Topic 4th:	station 0.037361, radio 0.021366, channel 0.016035, contest 0.013985, listen 0.012344, offer 0.007013, servic 0.006603, local 0.006193, roll 0.006193, call 0.005782, fm 0.005372, pai 0.004962, goodbi 0.004552, suit 0.004142, include 0.004142, meanwhil 0.003732, clean 0.003322, car 0.003322, promotion 0.003322, award 0.002912
Topic 5th:	office 0.026614, plant 0.023117, city 0.020669, depart 0.018570, energy 0.016472, nuclear 0.013324, oil 0.010876, asbesto 0.010177, worker 0.009478, oper 0.008778, power 0.006680, six 0.005980, action 0.005980, cover 0.005631, defense 0.005631, reactor 0.005631, remove 0.005281, control 0.004581, date 0.004232, manage 0.004232

Some difference and common characteristics can be seen from Tables 2 and 3. First, most of the words in same column are related with each other, and these relations are helpful for text clustering more or less. The difference is that the retrieved concept relevance records in HOWNET are more close to each other apparently. In other words, they share same clear topic. Whereas the words in the same topic generated by LDA are latent: some of them are apparently related with each other, whereas for others, it may be difficult to say there are closely related. i.e., there share one latent topic. For example, in topic 4th, we can understand the terms like “listen”, “radio”, “fm”,”channel” and so on are closely related with each other. But for other words like “car”, “clean”, “call” and so on, things may be different.

### 6.3 The impact of feature selection on clustering results

**Fig 6:**
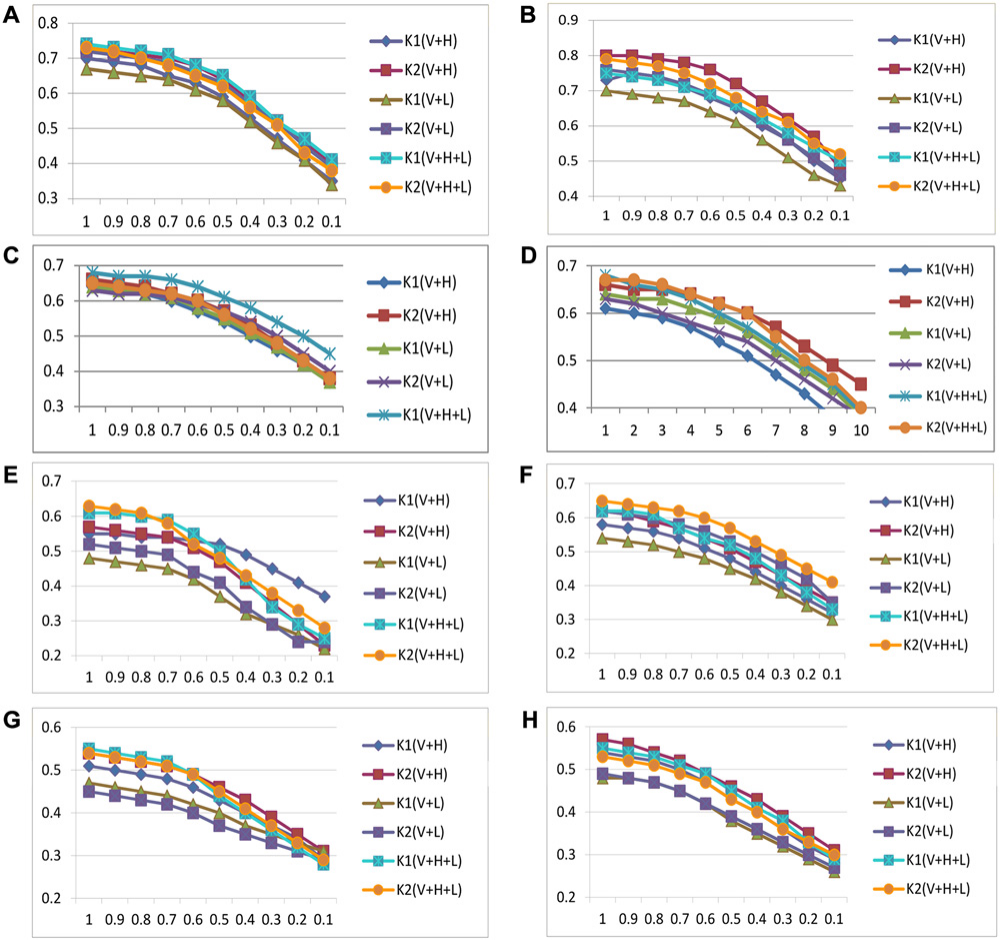
The impact of feature selection on clustering results. (A). Evaluation method: accuracy; Clustering method: k-means; Dataset: dataset 1; (B). Evaluation method: accuracy; Clustering method: SOM; Dataset: dataset 1; (C). Evaluation method: BF; Clustering method: k-means; Dataset: dataset 1; (D): Evaluation method: BF; Clustering method: SOM; Dataset: dataset 1; (E). Evaluation method: accuracy; Clustering method: k-means; Dataset: dataset 2; (F). Evaluation method: accuracy; Clustering method: SOM; Dataset: dataset 2; (G). Evaluation method: BF; Clustering method: k-means; Dataset: dataset 2; (H). Evaluation method: BF; Clustering method: SOM; Dataset: dataset 2.

The objective of text clustering is to put closed related documents into same cluster, and dissimilar documents into different clusters. We also find that if only some part of features is used, the clustering results may be not affected adversely. In Fig. 6, we examined the impact of feature selection on clustering results.

In Figs. 6(A)–(H), the X-axis is the feature selection percentage. It can be seen that if the percentage is above 0.7, i.e. from 1.0 to 0.7, the clustering results varies slowly, which means some salient features and the corresponding extension features may be sufficient for text clustering and the clustering quality loss is not too much.

Besides, as we use all words (after stop words removal; α = 0.8) in these figures, we can also see the performance comparison with the clustering results of the document-level extension. Generally, compared with all words are used (percentage is 1.0), about 0.3–0.5 higher clustering results can be achieved by using the document-level extension.

### 6.4 The comparison of time consumed between with and without semantic extension

In order to evaluate the clustering efficiency after semantic extension, we also make comparison of time consumed between with and without semantic extension. The comparisons of the average run time results in seconds, with or without semantic extension (Hownet-based), are shown in Tables 4 and 5.

**Table 4:** Comparison of time consumed with or without semantic extension on dataset 1 (: S; *-A: without extension; *-B: with extension).

K	K-means-A	K-means-B	SOM-A	SOM-B
K = 10	22.06	36.49	29.63	38.26
K = 50	39.82	52.83	43.29	56.31

**Table 5:** Comparison of time consumed with or without semantic extension on dataset 2 (: S: *-A: without extension; *-B: with extension).

K	K-means-A	K-means-B	SOM-A	SOM-B
K = 8	685.32	1335.02	776.36	1493.21
K = 20	2219.84	3468.35	2637.98	4137.24

Each experiment is performed ten times, and the average run time results are reported. The run time experiments were performed on a PC with an Intel Pentium IV 2.4 GHz CPU, 1 GB memory, and Windows XP operating system. Here we fixed the *k* value to be the same as the true number of classes in each data set, but different *k* values do not change our conclusion. Combined with the above table, we see that in order to better grasp the common semantic features of documents for vary-granularity text clustering, extra computational time will indeed be necessary. With regard to clustering quality and clustering efficiency, the former seems much more important because clustering results that cost less time but have poor clustering quality are not very useful. In applications that high clustering quality is preferred, our approach may be helpful.

## Conclusions and Future Work

In this paper, we have investigated an efficient feature modeling method for semantically sensitive text clustering and have tested its performance using a few existing text clustering algorithms. The feature quantization method, which codes documents by semantic extension technology, is able to discover the common features of documents; therefore, it is useful in vary-granularity text clustering applications. Similarity calculations based on our semantic extension strategy have been proven to have a more significant effect on clustering quality due to its ability to capture the common features hidden by complex language phenomena. We have also shown that a combination with the traditional VSM method is necessary, and our approach is compatible with traditional methods.

Although similarity computations in the extension space require extra time, in terms of the high-quality clustering results achieved and the actual text clustering effect of varied-granularity, it is an acceptable trade-off. In fact, semantic analysis may be accomplished in a preprocess step, and the number of dimensions in the extension space is much lower than that in the traditional space, thus the online clustering process need not cost an excessive amount of additional time and will be relatively economical for vary-granularity text clustering. Besides, fast clustering methods including efficient similarity computation and incremental clustering are compatible with our approach. Therefore, the time consumption will be further reduced.

There are a number of future research directions to extend and improve this study. One direction that this study may follow is to improve the accuracy of the similarity calculations by more finely utilizing a knowledge base and/or by updating the knowledge base through statistical processing. Although the current scheme appears to be more accurate and more sensitive to semantics than traditional methods, there is still room for improvement.
